# Decreased Levels of EGF in Plasma of Children with Autism Spectrum Disorder

**DOI:** 10.1155/2012/205362

**Published:** 2012-03-05

**Authors:** Charity Onore, Judy Van de Water, Paul Ashwood

**Affiliations:** ^1^Department of Medical Microbiology and Immunology, University of California, Davis, USA; ^2^The M.I.N.D. Institute, University of California, Davis, Sacramento, CA 95817, USA; ^3^Division of Rheumatology, Allergy and Clinical Immunology, University of California, Davis, USA

## Abstract

Autism Spectrum Disorder (ASD) is a neurodevelopmental disorder estimated to affect 1 in 110 children in the U.S., yet the pathology of this disorder is not fully understood. Abnormal levels of several growth factors have been demonstrated in adults with ASD, including epidermal growth factor (EGF) and hepatocyte growth factor (HGF). Both of these growth factors serve important roles in neurodevelopment and immune function. In this study, concentrations of EGF and HGF were assessed in the plasma of 49 children with ASD aged 2–4 years old and 31 typically developing controls of a similar age as part of the Autism Phenome Project (APP). Levels of EGF were significantly reduced in the ASD group compared to typically developing controls (*P* = 0.003). There were no significant differences in HGF levels in young children with ASD and typically developing controls. EGF plays an important role in regulating neural growth, proliferation, differentiation and migration, and reduced levels of this molecule may negatively impact neurodevelopment in young children with ASD.

## 1. Introduction

Autism Spectrum Disorder (ASD) is a developmental disorder characterized by impairments in social interaction and communication, and the presence of restricted behaviors or interests [1]. According to the most current CDC estimate, ASD affects 1 in 110 children in the US [2], yet the pathophysiology of the disorder is largely unknown. Recently, several growth factors have been found to be dysregulated in a substantial proportion of adults with ASD [3].

In the central nervous system, growth factors can regulate the processes of neuronal growth, differentiation, and proliferation, as well as regulating neuronal survival, neuronal migration, and the formation or elimination of synapses [3]. In addition to their central function in regulating neurodevelopment, current literature has also illustrated the dual nature of many growth factors as immune modulators and highlighted their involvement in crosstalk between the immune system and the central nervous system (CNS) [4–7]. Many studies suggest the presence of aberrant immune activity in ASD, in the CNS [8, 9] and in the periphery [10–12], which may be influenced by atypical growth factor activity. Growth factor dysregulation may contribute to ASD pathology by directly affecting CNS development, and/or by augmenting immune function.

Epidermal growth factor (EGF) and hepatocyte growth factor (HGF) are both involved in the growth and proliferation of several cell types, including neurons and glia of the CNS. EGF is present at high levels in the central nervous system (CNS) and plays a critical role in controlling proliferation and differentiation of nervous tissue during neurogenesis [13, 14]. In addition to the function of EGF as a CNS growth factor, it is a central factor in promoting wound healing. EGF is expressed at sites of injury and inhibits the activity of nitric oxide synthase, preventing inflammation [4, 15]. EGF deficiency results in several neurological, gastrointestinal, dermal, and pulmonary abnormalities in animal models [16]. An increased frequency of EGF single nucleotide polymorphisms has been reported in ASD, as well as lower plasma EGF levels in adults with autism [17, 18].

HGF also plays a dual role as a neurological growth factor and an immune modulator. HGF can modulate immune responses by signaling through the MET receptor on antigen presenting cells, which results in a tolerogenic phenotype with reduced proinflammatory cytokine production and cellular activity [5]. HGF is also essential for normal neurodevelopment, and disruption of HGF signaling results in complex alterations in GABAergic neuron development in the forebrain of animal models [19]. Lower levels of HGF in sera of autistic adults have been described [20], as well as decreased expression of the HGF receptor in postmortem brain samples [21].

To determine if there exists a differential profile for peripheral blood growth factor levels in ASD, we analyzed plasma EGF and HGF in well-characterized young children aged 2–4 years old with a diagnosis of ASD and unrelated typically developing children who were frequency-matched for age. In addition, levels of plasma EGF and HGF were investigated for any associations with clinical behavioral and developmental outcomes.

## 2. Methods and Materials

### 2.1. Subjects and Behavioral Assessments

 Eighty study participants aged between 2–4 years of age were recruited as part of the APP [22]. Participants consisted of 49 children with ASD (median age of 2.88 years, interquartile range 2.66–3.41 years, 42 males) and 31 typically developing (TD) children (median age of 2.96 years, interquartile range 2.85–3.27, 20 males). Diagnostic instruments included the Autism Diagnostic Observation Schedule-Generic (ADOS-G) [23] and the Autism Diagnostic Interview-Revised (ADI-R) [24]. All diagnostic assessments were conducted or directly observed by trained, licensed clinical psychologists who specialize in autism and had been trained according to research standards for these tools. Inclusion criteria for ASD were taken from the diagnostic definition of ASD in young children formulated and agreed upon by the Collaborative Programs of Excellence in Autism. Inclusion criteria for TD controls included developmental scores within two standard deviations of the mean on all subscales of the MSEL. Exclusion criteria for TD controls included a diagnosis of mental retardation, pervasive developmental disorder or specific language impairment, or any known developmental, neurological, or behavioral problems. TD children were screened and excluded for autism with the Social Communication Questionnaire (scores > 11) (SCQ—Lifetime Edition) [25].

### 2.2. Measurement of EGF

 Peripheral blood was collected in acid-citrate-dextrose Vacutainers (BD Biosciences, San Jose, CA). The plasma fraction was immediately harvested by centrifugation and stored as aliquots at −80°C until the date of assay. Plasma levels of EGF were determined by Human EGF enzyme-linked immunosorbant assay (ELISA) kit (R&D Systems, Minneapolis, MN). Plasma levels of HGF were determined by Human HGF enzyme-linked immunosorbant assay (ELISA) kit (R&D Systems). The assays were performed according to the protocols provided by the manufacturer, and all samples were assayed in duplicate. Optical density was measured on a Wallac Victor3 multilabel-plate reader (PerkinElmer, Boston, MA) at 450 nm.

### 2.3. Statistical Analysis

 Statistical analysis to compare levels of growth factors between ASD and TD groups was conducted with unpaired Student's *t*-test. All analyses were conducted with GraphPad Prism statistical software (GraphPad Software Inc., San Diego, CA).

## 3. Results

**Figure 1:**
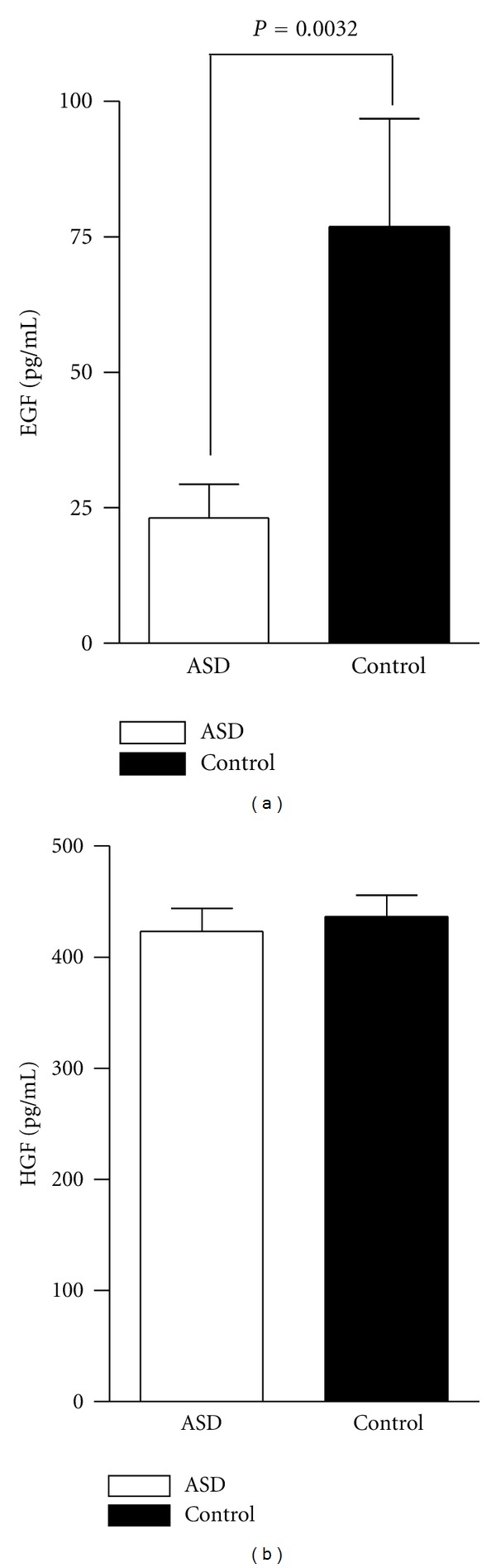
Plasma levels of EGF and HGF. Levels of EGF and HGF in peripheral blood plasma from ASD participants and similarly aged typically developing controls as measured by ELISA (data is shown as mean and SEM). EGF levels are significantly lower in ASD subjects in comparison to typically developing controls (*P* = 0.003). HGF levels did not differ significantly between the two groups. Significance was determined by two-tailed unpaired Student's *t*-test.

Plasma levels of EGF were approximately 3-fold lower in the ASD group (23.1 ± 6.2 pg/mL) compared with the TD group (76.9 ± 20.0 pg/mL) (*P* = 0.003). Plasma levels of HGF were not statistically different between children with ASD (423.5 ± 20.9 pg/mL) and TD controls (436.6 ± 19.2 pg/mL) (Figure 1).

## 4. Discussion

In this study, we investigated the levels of EGF and HGF, two growth factors involved in neurodevelopment. Previous studies have found decreased levels of EGF in high functioning adults with autism [18], but no studies have looked at children with ASD who are close to the onset of the disorder. We found that levels of plasma EGF were significantly reduced in young children with ASD as compared with similarly age-matched typically developing control children. This finding is consistent with that seen in adults with high functioning autism and may suggest that a deficiency in EGF is persistent throughout the time course of ASD.

Current research has demonstrated that EGF is involved in growth, differentiation, and maintenance of several tissues including the CNS and the gastrointestinal tract (GI) [14]. Notably, abnormalities of both the CNS and the GI have been reported in ASD [26]. In the CNS, EGF serves as a potent neurotrophic factor, and *in vivo* studies have demonstrated the effect of EGF in promoting proliferation, differentiation, survival, and migration of multipotent neural progenitor cells and the differentiation of these cells into astrocytes and neurons [27]. In this paper, we were limited to investigating EGF in plasma and not in cerebral spinal fluid. However, EGF rapidly transports across the blood-brain barrier [28], suggesting peripheral EGF levels could be representative of levels in the CNS that may impact neurodevelopment.

In the GI tract, EGF is necessary for normal development of the intestinal mucosa, and mice deficient in EGF receptor suffer from symptoms similar to those of necrotizing enterocolitis, with gradual destruction of villi, become severely malnourished, and typically die before postnatal day 8 [16]. EGF promotes wound healing in animal models of ulcerative colitis [29, 30]. Although GI symptoms affect a large proportion of children with ASD, the exact nature and extent of GI inflammation in ASD are still controversial [31–34]. Interestingly, preliminary investigations in this study show that EGF levels are associated with increased bloating in children with ASD, as well as with raw, standard, and percentile rank scores on the Peabody picture vocabulary test-III and with the composite and nonverbal development quotient of the Mullen's test (*P* < 0.05, data not shown). However, the meaning of this data is not clear and we will validate these findings in a larger replication cohort.

It has been previously reported that HGF was decreased in the plasma of adults with autism [20]; however, we did not find a significant difference between children with ASD and typically developing children for plasma HGF levels. This may be due to age differences between the participants of our study and those in the previous reports. To our knowledge, the relationship between age and serum HGF levels has not been thoroughly established, but there is evidence that levels may decrease with age [35].

Collectively, our data suggest that reduced levels of EGF are present in ASD. The roles of EGF and ASD require further research to better elucidate the relationship between this potent growth factor and ASD pathophysiology.

## References

[1] APA (2000). *Diagnostic and Statistical Manual of Mental Disorders*.

[2] Rice C (2009). Prevalence of autism spectrum disorders—autism and developmental disabilities monitoring network, United States, 2006. *Morbidity and Mortality Weekly Report*.

[3] Nickl-Jockschat T, Michel TM (2011). The role of neurotrophic factors in autism. *Molecular Psychiatry*.

[4] Heck DE, Laskin DL, Gardner CR, Laskin JD (1992). Epidermal growth factor suppresses nitric oxide and hydrogen peroxide production by keratinocytes. Potential role for nitric oxide in the regulation of wound healing. *Journal of Biological Chemistry*.

[5] Okunishi K, Dohi M, Nakagome K (2005). A novel role of hepatocyte growth factor as an immune regulator through suppressing dendritic cell function. *Journal of Immunology*.

[6] Vega JA, García-Suárez O, Hannestad J, Pérez-Pérez M, Germanà A (2003). Neurotrophins and the immune system. *Journal of Anatomy*.

[7] Tabakman R, Lecht S, Sephanova S, Arien-Zakay H, Lazarovici P (2004). Interactions between the cells of the immune and nervous system: neurotrophins as neuroprotection mediators in CNS injury. *Progress in Brain Research*.

[8] Vargas DL, Nascimbene C, Krishnan C, Zimmerman AW, Pardo CA (2005). Neuroglial activation and neuroinflammation in the brain of patients with autism. *Annals of Neurology*.

[9] Li X, Chauhan A, Sheikh AM (2009). Elevated immune response in the brain of autistic patients. *Journal of Neuroimmunology*.

[10] Ashwood P, Krakowiak P, Hertz-Picciotto I, Hansen R, Pessah IN, Van de Water J (2011). Altered T cell responses in children with autism. *Brain, Behavior, and Immunity*.

[11] Enstrom AM, Onore CE, Van de Water JA, Ashwood P (2010). Differential monocyte responses to TLR ligands in children with autism spectrum disorders. *Brain, Behavior, and Immunity*.

[12] Ashwood P, Krakowiak P, Hertz-Picciotto I, Hansen R, Pessah I, Van de Water J (2011). Elevated plasma cytokines in autism spectrum disorders provide evidence of immune dysfunction and are associated with impaired behavioral outcome. *Brain, Behavior, and Immunity*.

[13] Xian CJ, Zhou XF (1999). Roles of transforming growth factor-*α* and related molecules in the nervous system. *Molecular Neurobiology*.

[14] Xian CJ, Zhou XF (2004). EGF family of growth factors: essential roles and functional redundancy in the nerve system. *Frontiers in Bioscience*.

[15] Pastore S, Mascia F (2008). Novel acquisitions on the immunoprotective roles of the EGF receptor in the skin. *Expert Review of Dermatology*.

[16] Miettinen PJ, Berger JE, Meneses J (1995). Epithelial immaturity and multiorgan failure in mice lacking epidermal growth factor receptor. *Nature*.

[17] Toyoda T, Nakamura K, Yamada K (2007). SNP analyses of growth factor genes EGF, TGF*β*-1, and HGF reveal haplotypic association of EGF with autism. *Biochemical and Biophysical Research Communications*.

[18] Suzuki K, Hashimoto K, Iwata Y (2007). Decreased serum levels of epidermal growth factor in adult subjects with high-functioning autism. *Biological Psychiatry*.

[19] Levitt P, Eagleson KL, Powell EM (2004). Regulation of neocortical interneuron development and the implications for neurodevelopmental disorders. *Trends in Neurosciences*.

[20] Sugihara G, Hashimoto K, Iwata Y (2007). Decreased serum levels of hepatocyte growth factor in male adults with high-functioning autism. *Progress in Neuro-Psychopharmacology and Biological Psychiatry*.

[21] Campbell DB, D’Oronzio R, Garbett K (2007). Disruption of cerebral cortex MET signaling in autism spectrum disorder. *Annals of Neurology*.

[22] Singer E (2005). “Phenome” project set to pin down subgroups of autism. *Nature Medicine*.

[23] Lord C, Rutter M, Goode S (1989). Autism diagnostic observation schedule: a standardized observation of communicative and social behavior. *Journal of Autism and Developmental Disorders*.

[24] Lord C, Rutter M, Couteur AL (1994). Autism diagnostic interview-revised: a revised version of a diagnostic interview for caregivers of individuals with possible pervasive developmental disorders. *Journal of Autism and Developmental Disorders*.

[25] Berument SK, Rutter M, Lord C, Pickles A, Bailey A (1999). Autism screening questionnaire: diagnostic validity. *British Journal of Psychiatry*.

[26] Careaga M, Van de Water J, Ashwood P (2010). Immune dysfunction in autism: a pathway to treatment. *Neurotherapeutics*.

[27] Craig CG, Tropepe V, Morshead CM, Reynolds BA, Weiss S, Van Der Kooy D (1996). In vivo growth factor expansion of endogenous subependymal neural precursor cell populations in the adult mouse brain. *Journal of Neuroscience*.

[28] Pan W, Kastin AJ (1999). Entry of EGF into brain is rapid and saturable. *Peptides*.

[29] Farrell RJ (2003). Epidermal growth factor for ulcerative colitis. *New England Journal of Medicine*.

[30] Sinha A, Nightingale J, West KP, Berlanga-Acosta J, Playford RJ (2003). Epidermal growth factor enemas with oral mesalamine for mild-to-moderate left-sided ulcerative colitis or proctitis. *New England Journal of Medicine*.

[31] Horvath K, Perman JA (2002). Autism and gastrointestinal symptoms. *Current Gastroenterology Reports*.

[32] Ashwood P, Anthony A, Pellicer AA, Torrente F, Walker-Smith JA, Wakefield AJ (2003). Intestinal lymphocyte populations in children with regressive autism: evidence for extensive mucosal immunopathology. *Journal of Clinical Immunology*.

[33] Ashwood P, Anthony A, Torrente F, Wakefield AJ (2004). Spontaneous mucosal lymphocyte cytokine profiles in children with autism and gastrointestinal symptoms: mucosal immune activation and reduced counter regulatory interleukin-10. *Journal of Clinical Immunology*.

[34] Ashwood P, Wakefield AJ (2006). Immune activation of peripheral blood and mucosal CD3+ lymphocyte cytokine profiles in children with autism and gastrointestinal symptoms. *Journal of Neuroimmunology*.

[35] Ramirez R, Hsu D, Patel A (2000). Over-expression of hepatocyte growth factor/scatter factor (HGF/SF) and the HGF/SF receptor (cMET) are associated with a high risk of metastasis and recurrence for children and young adults with papillary thyroid carcinoma. *Clinical Endocrinology*.

